# Supplementary material for: Synchronization transition in neuronal networks composed of chaotic or non-chaotic oscillators
Source: Sci Rep. 2018 May 30;8:8370. doi: 10.1038/s41598-018-26730-9 (PMC5976724; doi:10.1038/s41598-018-26730-9)
Supplement: Supplementary file 1 — Supplementary Figures [file 41598_2018_26730_MOESM1_ESM.pdf]

Synchronization transition in neuronal networks composed of chaotic or non-chaotic oscillators

Kesheng Xu[1], Jean Paul Maidana[1], Samy Castro[1], Patricio Orio[1,2*]

[1]Centro Interdisciplinario de Neurociencia de Valparaíso, Universidad de Valparaíso, Valparaíso, Chile.
[2] Instituto de Neurociencia, Facultad de Ciencias, Universidad de Valparaíso, Valparaíso, Chile.

* patricio.orio@uv.cl

Supplementary Information

S1-Fig. Synchronization transitions on the case of g_h/g_{sd} with same distribution of Firing Rate.

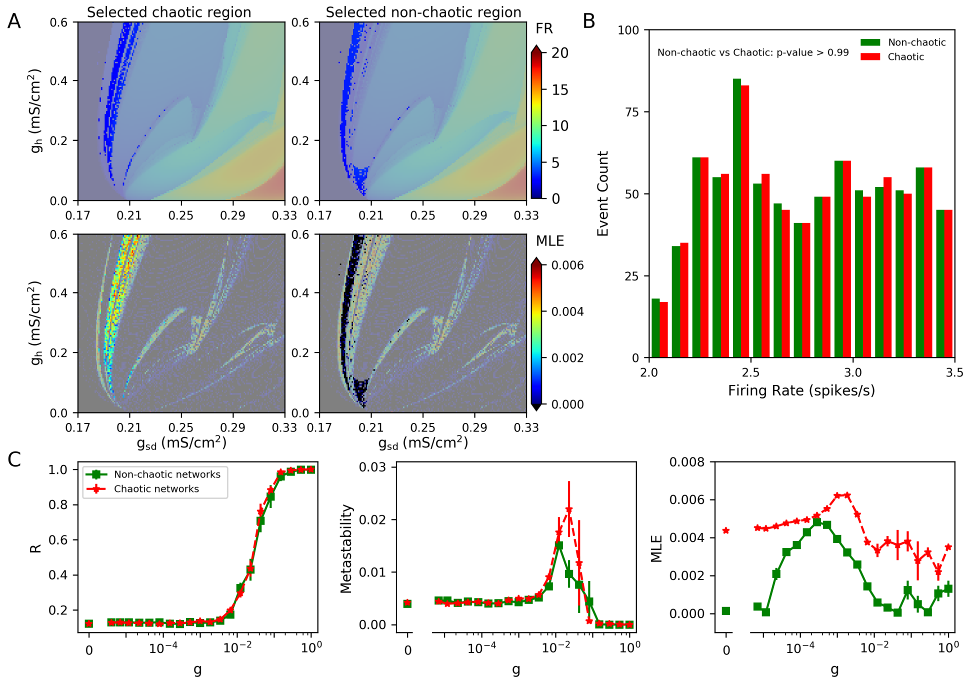

S1-Fig . Synchronization transitions on the neural networks with parameters chosen from same distribution of firing rate of g_{sd}/g_h parameter space. (A) FR (top) and MLE (bottom) obtained from each set of parameter values. The selected of blue regions in FR and MLE plot correspond to either chaotic (left) or non-chaotic oscillations (right). (B) Histogram of firing rates in each selected parameter regions, showing the same distribution of firing rate with ranges from 2.0 to 3.5 spikes/s. (C) Synchronization transition characterized by order parameter, metastability and the network MLE. The results obtained here are compatible with what has been plotted of main text (shown in **Fig 5** of main text).

S2-Fig. FCD in networks of chaotic and non-chaotic oscillators of the case shown in S1-Fig.

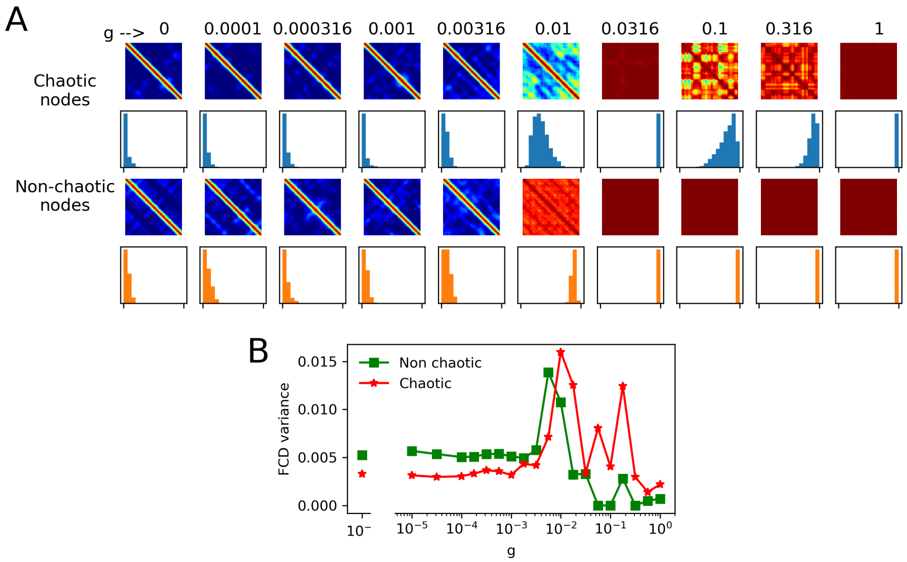

S2-Fig . FCD in networks of chaotic and non-chaotic oscillators. (A) FCD matrices obtained at different values of synaptic conductance g in networks of either chaotic or non-chaotic nodes. Below each matrix, an histogram of the values is shown. The diagonal and the neighboring values were not included. (B) Variance of the FCD values plotted against g. Average of 10 simulations with different random seed for the small-world connectivity and parameters.

S3-Fig. Network dynamics appears robust with respect to the network size of the same distribution of firing rates(g_{sd}/g_{sr}).

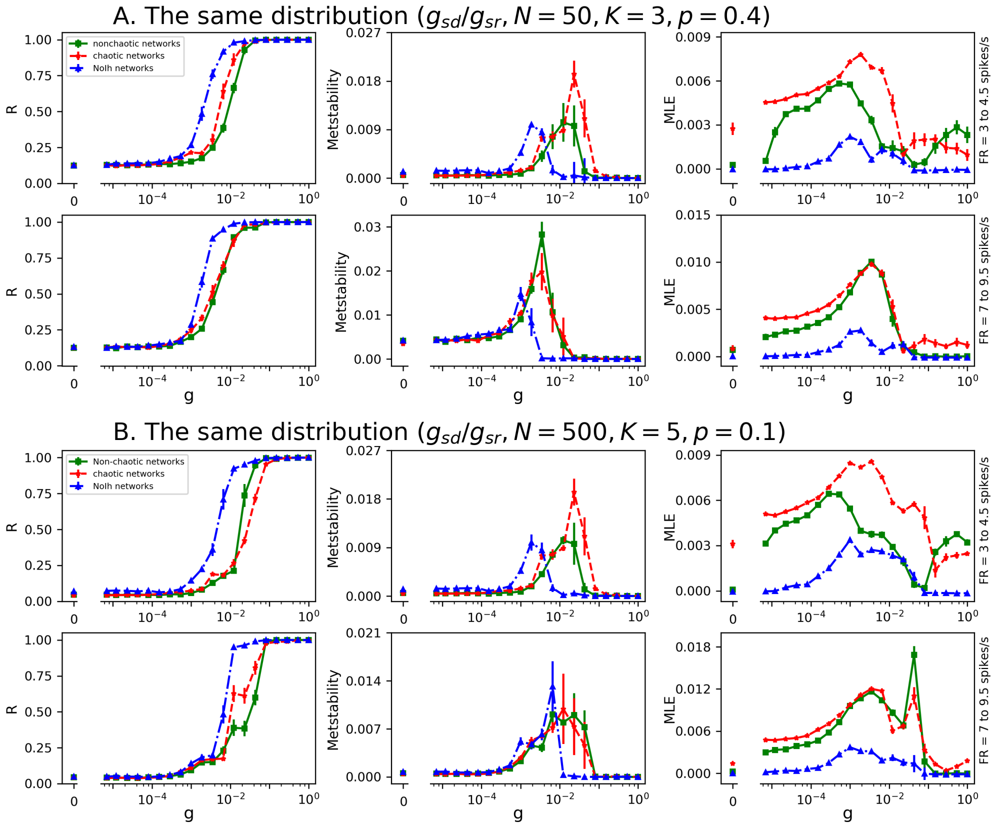

S3-Fig . Synchronization transitions are robust with respect to the network size of the same case as **Fig 4** of the main text. The network parameters are as follows: (A) $N = 50, K = 3, p = 0.4$ and (B) $N = 500, K = 5, p = 0.1$. Each curve is obtained over the average of 10 simulations with different random seeds for the small-world connectivity and parameters.

S4-Fig. FCD in networks composed of 50 and 500 neurons.

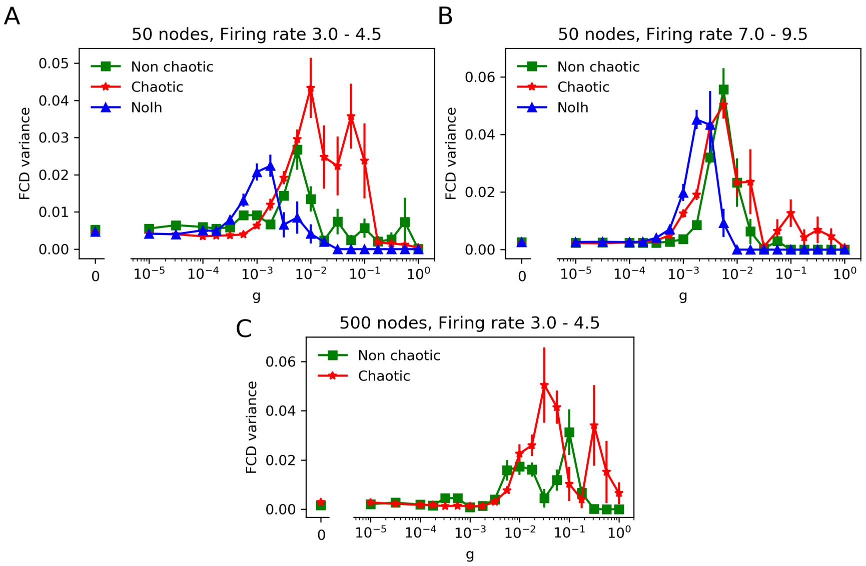

S4-Fig . Variance of the FCD values plotted against g, for networks of 50 (A, B) or 500 neurons (C) with the indicated ranges of firing rate. Average (±SEM) of 10 (A,B) or 6 (C) simulations with different random seeds for the small-world connectivity and parameters.
